# Enhanced Strength and Ductility in Magnesium Matrix Composites Reinforced by a High Volume Fraction of Nano- and Submicron-Sized SiC Particles Produced by Mechanical Milling and Hot Extrusion

**DOI:** 10.3390/ma12203445

**Published:** 2019-10-21

**Authors:** Sepideh Kamrani, Daniela Hübler, Alireza Ghasemi, Claudia Fleck

**Affiliations:** Department of Materials Engineering, Institute of Technology Berlin, 10623 Berlin, Germany; huebler@tu-berlin.de (D.H.); ghasemi@tu-berlin.de (A.G.); claudia.fleck@tu-berlin.de (C.F.)

**Keywords:** Mg–SiC nanocomposite, nano- and submicron-sized SiC particles, ultrafine structured, enhanced combination of strength and ductility

## Abstract

In the present study, Mg nanocomposites with a high volume fraction (10 vol %) of SiC particles were fabricated by two approaches: mechanical milling and mixing, followed by the powder consolidation steps, including isostatic cold pressing, sintering, and extrusion. A uniform distribution of the high content SiC particles in a fully dense Mg matrix with ultrafine microstructure was successfully achieved in the mechanically milled composites. The effect of nano- and submicron-sized SiC particles on the microstructure and mechanical properties of the nanocomposites was evaluated. Scanning electron microscopy (SEM), transmission electron microscopy (TEM), energy dispersive spectrometer (EDS), and X-ray diffractometry (XRD) were used to characterize microstructures of the milled and mixed composites. Mechanical behavior of the Mg composites was studied under nanoindentation and compressive loading to understand the effects the microstructural modification on the strength and ductility of the Mg/SiC composites. The mechanical properties of the composites showed a significant difference regarding the size and distribution of SiC particles in the Mg matrix. The enhanced strength and superior ductility achieved in the mechanically milled Mg composites are mainly ascribed to the effective load transfer between matrix and SiC particles, grain refinement of the matrix, and strengthening effects of the nano- and submicron-sized SiC particles.

## 1. Introduction

The combination of magnesium (Mg) with ceramic particles results in materials with innovative and multifunctional properties such as low density, high specific stiffness, strength, and damping capacity. However, while the addition of micron size ceramic particles improves strength significantly, it usually deteriorates the ductility due to the brittle nature of the particles. Many attempts have therefore been made to simultaneously increase strength and ductility of such composite materials to make these materials more suitable for applications where weight reduction is a critical factor, such as in automotive, aerospace, and sports domains [1,2,3]. Reducing the size of the ceramic particles to the submicron- or nano-meter regime is a promising strategy to produce composites with high strength and ductility [4,5,6,7], as has, for instance, been shown for Mg composites reinforced by 1.1 vol % Al_2_O_3_ nano- and submicron particles [8]. A significant increase in ductility from 4.2% to 12.0% was also observed when nano-Y_2_O_3_ particles (0.66 vol %) were added to pure Mg [9]. Ferguson et al. [10] collected the available experimental data for Mg nanocomposites in the reported literature and analyzed the contribution of the primary strengthening mechanisms of the nanoparticles, such as the Orowan strengthening and the grain refinement, in simultaneously improving both strength and ductility. They pointed out that the Mg nanocomposites that appeared to have these improvements were generally those with lower volume fractions, indicating that agglomeration is prevalent at higher volume fractions. When the volume fraction of nanoparticles is high, it is much more difficult to disperse them, because of their high tendency to agglomerate into large clusters. Although theoretically, nanoparticles have the potential to improve the mechanical properties of Mg composites significantly, paths toward making this a reality are needed.

Over recent years, high energy mechanical milling has been found to be a promising and relatively inexpensive method to overcome the agglomeration typically observed in nanocomposites [11,12,13,14,15]. However, during the mechanical milling process, severe strain hardening and microstructural refinement take place, and this severe plastic deformation impedes further plastic deformation of the composite particles during the subsequent compaction stages. Therefore, milled powders consolidated by conventional compaction methods, such as uniaxial pressing and sintering, usually revealed a high level of porosity and more irregular pores with a broad pore size distribution, especially for high volume fractions of reinforcing particles [16]. By a well-defined processing strategy, where we combined mechanical milling of composite powders with adapted consolidation steps comprising a sequence of isostatic cold pressing, sintering, and hot extrusion, we recently managed to produce Mg matrix nanocomposites with a uniform distribution of SiC nanoparticles up to a high volume fraction of 10 vol % of the reinforcing phase and with a very high relative density of up to 99.9% [17].

Here, we report on the mechanical properties of these mechanically milled Mg composites and compare them to the properties of composites made by conventional mixing. Particular emphasis is given to the effect of the mechanical milling process on achieving a simultaneous improvement in strength and ductility for Mg nanocomposites with a high volume fraction of SiC nanoparticles of 10 vol %. We combined microstructural characterization by X-ray diffraction, optical, scanning, and transmission electron microscopy with mechanical analysis by nanoindentation and compression testing, and we deduced correlations between microstructure and mechanical properties. Lastly, we describe the damage mechanisms. 

## 2. Materials and Methods

### 2.1. Materials

Mg nanocomposites were processed according to the protocols described in detail previously [17,18,19]. In a nutshell, Mg powder with an average particle size of −325 mesh and nano- and submicron-sized SiC particles with an average particle size of 50 nm and ˂1 µm, respectively, (both Alfa Aesar, Ward Hill, MA, USA) were used. Mg powders with 10 vol % of nano- and submicron-sized SiC particles were mechanically milled up to 10 h to produce Mg–SiC nanocomposite and submicrocomposite powders (M10S_n_ and M10S_µ_, respectively). The milled composite powders were cold isostatically pressed at a pressure of 700 MPa with a holding time of 10 min, sintered at 600 °C for 2 h in argon atmosphere, and further densified through hot extrusion at 400 °C with a press ratio of 22:1. For each step, parameters were adjusted to achieve the highest possible density and fully dense nanocomposites at the end of the processing chain. As a reference, a Mg–10 vol % SiC_n_ powder mixture (named M10S_n_-mixed) that was not mechanically milled was processed by the same consolidation steps. The structure and material characteristics of the extruded composite materials are reported in Table 1.

Microstructural features of the consolidated bulk composites during the different processing steps were analyzed by high resolution scanning electron microscopy equipped with backscatter and in-lens detectors (HRSEM, Gemini 500, Zeiss, Germany). Elemental analysis was performed by energy dispersive X-ray spectrometry (EDX, Bruker Quantax, Berlin, Germany) in the HRSEM. 

**Figure 1:**
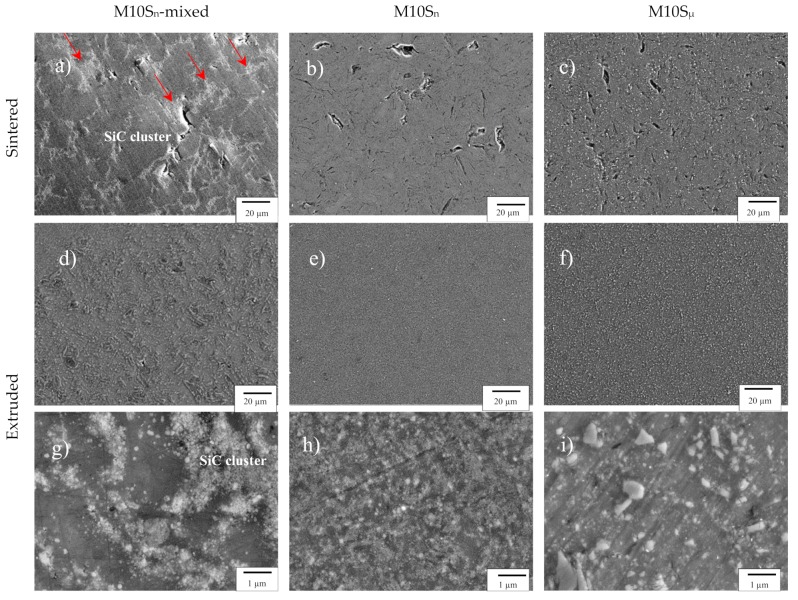
SEM micrographs of sintered (**a**–**c**), low (**d**–**f**), and high (**g**–**i**) magnification of hot extruded M10S_n_-mixed, M10S_n_, and M10S_µ_ composites.

Figure 1 shows the microstructures of the investigated composites after sintering and after hot extrusion. The SEM micrographs of the M10S_n_-mixed nanocomposite in the sintered state revealed extensive agglomeration of the SiC nanoparticles in big clusters (Figure 1a). Even by hot extrusion with a high extrusion ratio of 22:1, it was not possible to break up these clusters, and only a low degree of homogenization was achieved (Figure 1d,g). In contrast, both mechanically milled composites showed a very homogeneous microstructure, and, most importantly, particle agglomeration was avoided for the nanocomposite. No micrometer-sized particle clusters were observed (Figure 1e,f,h,i). Independent of the reinforcement size, hot extrusion closed the majority of the pores and the microcracks still present in the sintered samples. It is, therefore, a crucial step to achieve nearly full density in all samples. The relative densities of the samples after sintering and hot extrusion were determined in ethanol by the Archimedes method, and the values after hot extrusion are summarized in Table 1. 

The reinforcement distribution and the grain structure of the Mg matrix were evaluated qualitatively by transmission electron microscopy (TEM, Tecnai G2 20 S-TWIN, FEI, Hillsboro, OR, USA), on specimens prepared by the focused ion beam (FIB) technique. The statistical grain size distribution was estimated from several dark-field micrographs, in which around 100 grains were evaluated. X-ray diffraction (XRD) analysis was carried out in a Panalytical X‘Pert Pro diffractometer (Panalytical, Almelo,, The Netherlands) with Cu–Ka radiation between 20° and 80° to determine the grain size of the Mg matrix and the number and nature of the phases present after extrusion. The average grain sizes of the Mg matrix of the extruded composites based on the refinement of the XRD patterns and the TEM results are listed in Table 1. Texture was determined using a psi diffractometer (Huber, Rimsting, Germany) by measuring incomplete pole figures used monochromatic Co K_α_ radiation to measure the intensity distribution of the {$10\bar{1}0$}, {0002}, {$10\bar{1}1$}, {$10\bar{1}2$}, and {$11\bar{2}0$} reflections by tilting psi from 0° to 55° and rotating phi from 0° to 355° in 5 deg steps.

### 2.2. Mechanical Characterization

The mechanical properties of the composite samples after hot extrusion were investigated by compression, Vickers microhardness, and nanoindentation tests. For the quasi-static uniaxial compression test, cylindrical specimens with a diameter of 5 mm and a height of 10 mm were cut in the extrusion direction by wire electric discharge machining (EDM, GF Agie Charmilles, Losone, Switzerland). The surfaces were polished down to 1 µm to remove the oxide and EDM-induced damage surface and to generate smooth surfaces. The tests were performed at room temperature in ambient air with a strain rate of 10^−4^ s^−1^. After compression until failure, the specimens were cut parallel to the compression axis and the sections were observed by HRSEM to characterize the microstructure deformation and damage modes. Microhardness measurements were obtained using a Vickers microhardness tester (Zwick 3212, Ulm, Germany) with a force of 1.961 N (HV0.2) and a dwell time of 10 s.

For a deeper understanding of the nano- and microstructural parameters of the mechanical properties, nanoindentation measurements were performed at room temperature with a nanoindenter (Hysitron TI950 TriboIndenter, Bruker, Minneapolis, MN, USA) equipped with a standard Berkovich diamond indenter tip. The maximum load was 1 mN, both the loading and unloading rates were 200 μN s^−1^, and the dwell time at maximum load was 10 s. For each sample, at least twenty indentations were performed with a minimum distance of 20 µm. The shear band formation, damage, pile-up, and sink-in of materials around the contact area were studied deeply.

The indentation nanohardness (*H_n_*) is expressed as:

(1) $$H_{n}=\frac{P_{max}}{A_{c}}$$

where *A_c_* is the contact area at the maximum load and *P_max_* is the maximum normal load.

The contact stiffness (*S*) was measured continuously from the load displacement as the slope of the upper portion of the unloading curve. The elastic modulus of the material, E, was then derived from the contact stiffness following the standard Oliver and Pharr method [20] with

(2) $$E_{r}=\frac{\sqrt{\pi }}{2}\frac{S}{A_{c}}$$

and

(3) $$\frac{1}{E_{r}}=\frac{1-v^{2}}{E}+\frac{1-v_{i}^{2}}{E_{i}}$$

where *E_r_* is the reduced elastic modulus, which represents the elastic deformation that occurs in both sample and indenter tip; *E* and *v* are the elastic modulus and Poisson’s ratio, respectively, of the test material; and *E_i_* and *v_i_* are the elastic modulus and Poisson’s ratio, respectively, of the indenter material.

## 3. Results

### 3.1. Quasi-Static Compressive Mechanical Response

**Figure 2:**
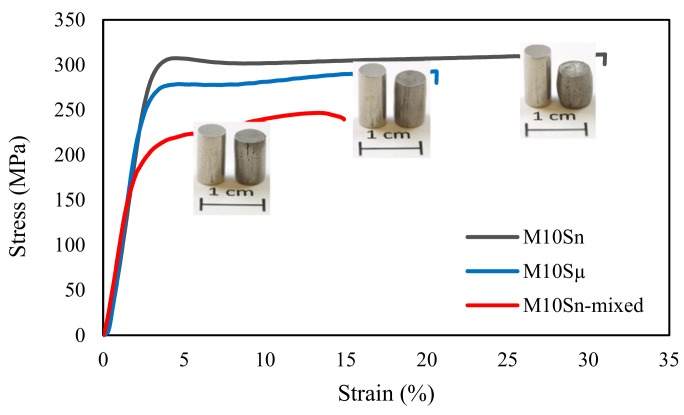
Compressive stress–strain curves of M10S_n_ M10S_µ_, M10S_n_-mixed composites with inserted macrographs the samples before and after compression test.

Typical compressive stress–strain curves of the M10S_n_, M10S_μ_, and M10S_n_-mixed specimens are presented in Figure 2. Compressive mechanical properties, including 0.2% compressive yield strength (0.2% CYS), ultimate compressive strength (UCS), and ultimate elongation of the Mg composites, were determined and are summarized in Table 2. The mechanically milled nanocomposite, M10S_n_, showed a great enhancement, not only in strength but also in elongation, as compared to the mechanically milled submicrocomposite, M10S_μ_. This suggests that, although the plasticity is not reduced by SiC nanoparticle addition, the compressive strength can be retained for prolonged periods. The simultaneous increase in strength and ductility of the M10S_n_ is also outstanding when compared to the mixed nanocomposite (M10S_n_-mixed). The M10S_n_ showed nearly 83%, 18%, and 138% enhancement in the 0.2% CYS, UCS, and ultimate elongation, as compared to the values for the M10S_n_-mixed composite, respectively. The mechanical characterization results revealed an excellent agreement between microhardness and compressive strength of the composites.

**Figure 3:**
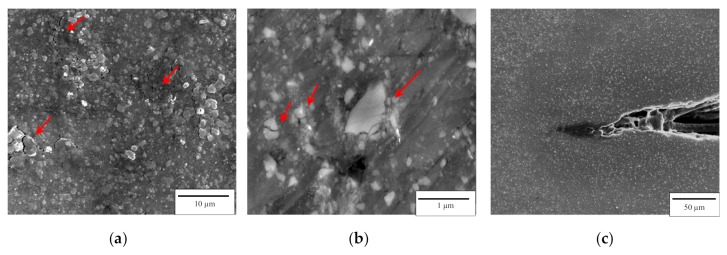
(**a**) SEM micrograph showing microcracks at 10% strain, (**b**) HRSEM micrograph showing broken submicron SiC particles at 20% strain in the compression-deformed M10S_µ_ composite, and (**c**) SEM micrograph showing a cracking path within the microstructure of M10S_µ_ at 20% strain.

During the quasi-static compression test, the events of microcrack initiation and propagation aligned with the compression direction were monitored with the help of the SEM analysis. Figure 3 shows the microstructure of the compression-deformed M10S_µ_ submicrocomposite at the strain of 10% and 20% (ultimate elongation). It is possible to observe evidence of microcracking when deformed past 10% strain, as indicated by arrows in Figure 3a. HRSEM analysis of the submicrocomposite at a higher magnification is shown with a high fraction of cracked particles (indicated by arrows), this was almost neglected after extrusion. However, no evidence of cracking in SiC nanoparticles was observed in the M10S_n_ nanocomposite during the compression deformation.

### 3.2. Quasi-Static Nanoindentation Response

**Figure 4:**
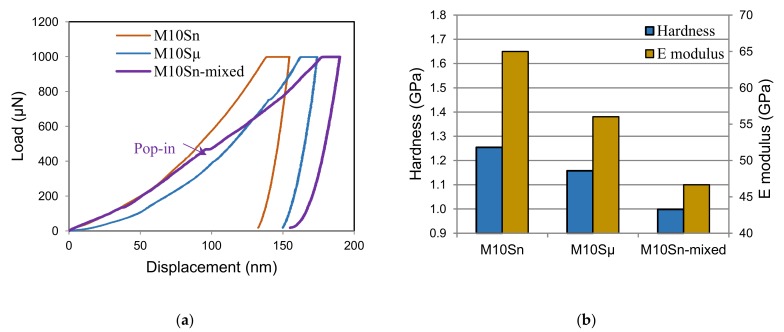
(**a**) Load-displacement curves obtained from nanoindentation experiments with a maximum load of 1 mN and (**b**) mean nanohardness and elastic modulus of M10S_n_, M10S_µ_, M10S_n_-mixed composites.

Figure 4 shows the results of the nanoindentation tests. The load-displacement curves show an approximately parabolic shape for the loading part and a very steep unloading part, which is linear down to very low loads. These typical graphs indicate an elastic-plastic behavior of the composites with relatively small elastic strains as compared with the plastic strains. Correspondingly, there was a very low amount of elastic recovery after unloading (Figure 4a). While the loading–unloading curves have a smooth course for the submicro- and nanocomposites made from mechanically milled powders (M10S_µ_, M10S_n_), a pop-in effect (a sudden increase in displacement at constant load) appears in the loading curves of the nanocomposite made from the mixed powder (M10S_n_-mixed). Furthermore, the load-displacement curves shifted to the left and maximum depth was reduced through the mechanical milling and addition of SiC nanoparticles. As a result, the measured nanohardness and elastic modulus of the M10S_n_ nanocomposite were significantly higher than the values obtained for M10S_µ_ and M10S_n_-mixed composites (Figure 4b). For instance, the nanohardness and the elastic modulus of the M10S_n_ nanocomposite enhanced up to 30% and 38%, respectively, as compared to the M10S_n_-mixed nanocomposite. The elastic modulus of the M10S_n_ composite was remarkably close to the computed elastic modulus using the rule of mixture (>70 GPa).

**Figure 5:**
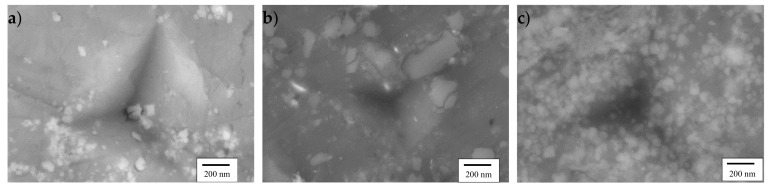
HRSEM micrographs of typical nanoindents on (**a**) M10S_n_-mixed; (**b**) M10S_µ_; and (**c**) M10S_n_ composites.

Figure 5 shows typical SEM micrographs of indents with the same maximum load of 1 mN on the extruded M10S_µ_, M10S_n_, and M10S_n_-mixed composites. Neither the indented surfaces nor the surfaces surrounding the indents showed any signs of cracking in the Mg matrix, highlighting the capability of the composites to deform plastically. It can be observed that the M10S_n_ exhibited the smallest indent and the least penetration depth compared to the rest of the samples, which can be attributed to its higher hardness and greater resistance to indentation. The HRSEM images of the locally deformed region in the M10S_n_-mixed composite revealed interfacial debonding between the Mg matrix and the SiC nanoparticles (the clusters and even individual particles) (Figure 5a). The SiC particles with submicron sizes were easy to crack or debond from the surrounding Mg matrix (Figure 5b). However, bonding and cohesion between the Mg matrix and the SiC nanoparticles seemed to be much stronger in the milled M10S_n_ composite, as no interfacial debonding was observed. Additionally, these observations provided no evidence of particle cracking in the SiC nanoparticles during the loading and unloading stages of the indentation test.

## 4. Discussion

The results of mechanical properties determined at the macro- and nanoscale show that the mechanical milling process, as well as the addition of nanoparticles, can simultaneously improve both the strength and the elongation of Mg matrix composites reinforced by a high volume fraction of nanometer-sized particles. Characterization of the nano- and microstructural features of the Mg composites before and after mechanical loading provides a deeper understanding of mechanisms resulting in the high mechanical strength and ductility.

The significant differences in the measured elastic modulus of the produced Mg composites with the same SiC content (65, 56, 46 GPa for the M10S_n_, M10S_µ_, M10S_n_-mixed, respectively) reveal that in addition to adding high elastic SiC particles (~420 GPa), other features can have a significant effect on the elastic modulus of the composites. It has been reported by several studies that the dominant factors in controlling the elastic modulus of metal matrix composites are the volume fraction, size, distribution, shape, and type of the reinforcement particle [21,22]. The clustering of the reinforcement particles has been identified as a major cause of reducing the elastic modulus of composites. Plastic flow is often localized in the vicinity of particle cluster, which can effectively inhibit dislocation movement. Slipenyuk et al. has reported that a boundary between agglomerated particles does not transfer tensile and shear stresses, even if the boundary between clusters does not contain voids and does not contribute to the porosity of the material [23]. Here, the remarkably low elastic modulus of the mixed nanocomposite can mainly be ascribed to the nanoparticle clusters in the Mg matrix, which significantly decrease the effective volume fraction of the reinforcement. In addition, the debonding of the particle/matrix interface in the mixed nanocomposite (Figure 5a) is another possible intrinsic reason for the low elastic modulus, since interface debonding leads to crack initiation and propagation. Meanwhile, the uniformly distributed nanoparticles enhance the elastic modulus of the composite (41%, compared to the mixed one) by behaving like a homogeneous structure and resisting the plastic deformation at lower stress. The results also showed that the elastic modulus of the Mg composite was improved by decreasing the size of SiC particles from submicron-scale to nanoscale. This is mainly attributed to the higher interfacial surface area provided by the nanoparticles, which enhance the load transfer between the matrix and the SiC particles. Further, the greater tendency of particle cracking and fragmentation in the submicron SiC particles (Figure 5b) compared to the nanoparticles can result in the lower elastic modulus of the submicrocomposite.

The uniform incorporation of the SiC nanoparticles into the Mg matrix has resulted in significant improvements in the compressive strength of the nanocomposite. Several strengthening mechanisms concerning the developed microstructure in the nanocomposite have been utilized to improve the strengths. (i) Hall–Petch strengthening in the ultrafine grain sizes of the Mg matrix is considered to be a major strength enhancement contributor in the milled nanocomposite. The ultrafine Mg structure was achieved and remained due to the mechanical milling and pinning effect of the SiC nanoparticles, respectively, as observed in our previous work [17]. (ii) The strong interfacial bonding between the Mg and SiC particles due to well-bonded and clean matrix reinforcement interfaces, formed during the mechanical milling process [18], allow effective load transfer from the Mg matrix to the SiC nanoparticles, leading to the improved strength. (iii) The presence and reasonable distribution of the high volume fraction of SiC nanoparticles in the Mg matrix provide strengthening in the milled nanocomposite due to Orowan strengthening. It is well-known that the contribution of the Orowan strengthening effect significantly decreases as particle size and interparticle distance increase [24]. An increase of interparticle distance in the M10S_n_-mixed nanocomposite due to the large numbers of the SiC nanoparticle clusters significantly decreased the Orowan effect for the nanocomposite. On the other hand, for the submicrocomposite, M10S_µ_, the Orowan effect was reduced with the increase in SiC particle size to submicron length scale. The lower amount of matrix/reinforcement interfaces, as well as coarser grain size of the Mg matrix reinforced with submicron SiC particles, compared to the SiC nanoparticles, diminished the load transfer and Hall-Petch strengthening mechanism, respectively.

**Figure 6:**
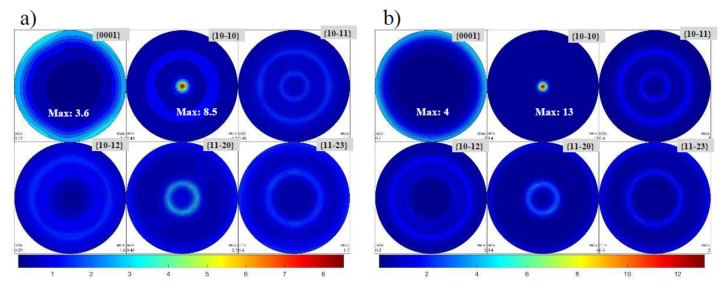
Basal, prismatic, and pyramidal pole figures of extruded (**a**) M10S_µ_ and (**b**) M10S_n_.

The nanoparticle clusters in the M10S_n_-mixed nanocomposite acted as preferred sites for microcrack or micro-void nucleation, which significantly reduce the ductility. A uniform distribution of the nanoparticles shows a good ability to increase the ductility of pure Mg compared to that of submicron particles. The revealed ability can be attributed to the combined effect of (i) the ultrafine grain structures of the Mg matrix, which reduce the size of the nucleating flaws and increase the resistance to crack propagation, leading to higher elongation; (ii) the reduced probability of particle cracking in the SiC nanoparticles during loading compared to that of submicron particles because the cracked particles can act as discontinuous flow localization, which helps initiate and propagate microcracks in the Mg matrix directly (Figure 3); and (iii) evolution of a weak basal texture. Based on the results shown in Figure 6, non-basal texture, especially prismatic slip, is likely to be developed in the M10S_µ_ and M10S_n_ composites (maximum texture intensity are 8.5 and 13, respectively), contrary to the strong basal texture which is typical seen in wrought Mg materials [25,26]. This observation makes a convincing case for particle-stimulated nucleation of dynamic recrystallization as a potential source mechanism for texture modification during hot processing [27,28]. The ultrafine SiC particles stimulate the dynamic recrystallization and create the non-basal texture in the Mg matrix, leading to the higher ductility. 

## 5. Conclusions

We studied the effect of nanometer- and submicron-sized SiC particles on the microstructure and mechanical properties of Mg–SiC nanocomposites processed by mixing and mechanical milling, and subsequently consolidated by isostatic cold pressing, sintering, and hot extrusion. The following were our main results:

- Mechanical milling can successfully prevent agglomeration of the SiC nanoparticles up to a high volume fraction of 10 vol %.
- The agglomeration of the SiC nanoparticles in the M10S_n_-mixed nanocomposite leads to poor compacting, sintering, and extrusion performance.
- Microstructural characterization of the locally deformed region through nanoindentation revealed interfacial debonding between the Mg matrix and nanoparticle clusters in the mixed nanocomposite, and particle fragmentation in the submicrocomposite.
- Compared with the mixed nanocomposite, a remarkable enhancement in the elastic modulus of the milled nanocomposite can be related to the homogeneous distribution of the nanoparticles and strong interface bonding between the nanoparticles and the Mg matrix developed during the mechanical milling process.
- Due to the strengthening effect from the ultrafine-grained Mg matrix and the uniform dispersions of the high volume fraction of SiC nanoparticles, the mechanically milled nanocomposite possessed prominent mechanical strengths.
- The remarkably enhanced ductility of the high content Mg–SiC nanocomposite was attributed to the activation of the non-basal slip systems that might be induced by dynamic recrystallization, but is dependent on the distribution of the SiC nanoparticles added.
- The produced ultrafine/nano Mg structure reinforced with uniform distribution of the SiC_n_ with a high volume fraction of 10 vol % had improved combinations of high strength and ductility, which show great promise for a variety of applications where lightweight materials are required.

## Figures and Tables

**Table 1:** SiC particle size, relative density, and average grain size of the extruded Mg composites.

Sample	Sic Particle Size	Milling Time, h	Relative Density, %	Grain Size, nm	
				XRD	TEM
M10S_n_	50 nm	10	99.4	126	155 ± 8
M10S_µ_	<1 µm	10	98.4	180	---
M10S_n_-mixed	50 nm	-	99.2	462	---

**Table 2:** Room temperature compressive mechanical properties of extruded Mg–SiC composites.

Materials	M10S_n_	M10S_µ_	M10S_n_-Mixed
0.2% CYS (MPa)	293 ± 2	246 ± 1	160 ± 2
UCS (MPa)	312 ± 3	292 ± 1	265 ± 6
Ultimate elongation (%)	32 ± 2	20 ± 2	13 ± 4
Microhardness (HV0.2)	99 ± 1	84 ± 6	
